# The Relationship between Type 1 Diabetes Mellitus, TNF-α, and IL-10 Gene Expression

**DOI:** 10.3390/biomedicines11041120

**Published:** 2023-04-07

**Authors:** Jesselina Francisco dos Santos Haber, Sandra Maria Barbalho, Jose Augusto Sgarbi, Rafael Santos de Argollo Haber, Roger William de Labio, Lucas Fornari Laurindo, Eduardo Federighi Baisi Chagas, Spencer Luiz Marques Payão

**Affiliations:** 1 School of Medicine, University of Marília (UNIMAR), Avenida Hygino Muzzy Filho, 1001, Marília 17525-160, Brazil; 2 Postgraduate Program of Health and Aging, Marilia Medical School (FAMEMA), Monte Carmelo, 800-Fragata, Marília 17519-030, Brazil; 3 Postgraduate Program in Structural and Functional Interactions in Rehabilitation, University of Marilia (UNIMAR), Marília 17525-160, Brazil; 4 Division of Endocrinology and Metabolism, Department of Medicine, Marilia Medical School (FAMEMA), Monte Carmelo, 800-Fragata, Marília 17519-030, Brazil; 5 Department of Genetics, Marilia Medical School (FAMEMA), Monte Carmelo, 800-Fragata, Marília 17519-030, Brazil

**Keywords:** gene expression, type 1 diabetes mellitus, interleukin 10, tumor necrosis factor-α, HbA1c, ketoacidosis

## Abstract

Type 1 diabetes mellitus (T1DM) is one of the major chronic diseases in children worldwide. This study aimed to investigate interleukin-10 (IL-10) gene expression and tumor necrosis factor-alpha (TNF-α) in T1DM. A total of 107 patients were included, 15 were T1DM in ketoacidosis, 30 patients had T1DM and HbA1c ≥ 8%; 32 patients had T1DM and presented HbA1c < 8%; and 30 were controls. The expression of peripheral blood mononuclear cells was performed using the reverse transcriptase–polymerase chain reaction in real time. The cytokines gene expression was higher in patients with T1DM. The IL-10 gene expression increased substantially in patients with ketoacidosis, and there was a positive correlation with HbA1c. A negative correlation was found for IL-10 expression and the age of patients with diabetes, and the time of diagnosis of the disease. There was a positive correlation between TNF-α expression with age. The expression of IL-10 and TNF-α genes showed a significant increase in DM1 patients. Once current T1DM treatment is based on exogenous insulin, there is a need for other therapies, and inflammatory biomarkers could bring new possibilities to the therapeutic approach of the patients.

## 1. Introduction

Type 1 diabetes mellitus (T1DM) is considered a chronic disease characterized by hyperglycemia and a broad spectrum of clinical manifestations [1] due to an absolute deficiency of insulin secretion [2,3]. This condition is determined by an autoimmune process resulting from a complex interaction between genetic and environmental factors and cytokine inflammatory pathophysiology [4,5]. The mechanisms involved in the development of this condition are related to a breakage in the central and peripheral tolerance, immune activation of T lymphocytes, and imbalance between Th1 and Th2 inflammatory response with the production of cytokines, leading to the progressive destruction of the β-cells and the gradual loss of insulin production [6,7,8].

IL-10 is known to exert profound and diverse anti-inflammatory effects, such as inhibiting pro-inflammatory cytokines and antigen-presenting cells, promoting tissue-repairing mechanisms, and playing an important role in restricting an excessive inflammatory response [9,10]. IL-10 plays a critical pleiotropic role in the immune system, maintaining the balance in the immune response necessary to defend the body and protect tissues during infection [11,12,13]. Thus, this cytokine influences the inflammatory response by inhibiting the effector function of Th1 cells [11]. Its activation is mediated by the IL-10 receptor (IL-10R), inhibiting the expression of class II major histocompatibility complex (MHC) and adhesion molecules, such as CD80 (B7.1), CD86 (B7.2), and CD54 [9,12]. It inhibits the expression of several inflammatory interleukins (TNF-α, IL-1β, IL-6, IL-8, G-CSF, and GM-CSF) and stimulates cell proliferation in B lymphocytes, preventing cell proliferation and apoptosis [14]. In this context, IL-10 exerts profound and diverse anti-inflammatory effects, such as inhibiting pro-inflammatory cytokines and antigen-presenting cells, promoting tissue repair mechanisms, and having an essential role in restraining an excessive inflammatory response [10]. However, the role of IL-10 in protecting β-cells during the pathogenesis and in the progression of T1DM remains controversial [9,14,15].

Additionally, TNF-α is a pro-inflammatory cytokine that stimulates IL-1, IL-6, fibroblast, and procoagulant factors and increases the expression of adhesion molecules [16]. It is produced by activated macrophages, dendritic cells, neutrophils, CD4+ lymphocytes, mast cells, eosinophils, neurons, and natural killers cells. It exerts this function by stimulating the production of IL-1β and IL-6 and increasing the expression of adhesion molecules, in addition to initiating the apoptotic and cytotoxic response [17,18,19].

Its role in DM1 is associated with other cytokines. This inflammatory process occurs after its release by activated CD4+ T cells, which leads to necrosis and local pancreatic lymphocytic infiltration, culminating in the death of the β cell and local stimulation of the pancreatic to produce TNF-α. Thus, it is directly involved in the damage caused to β cells and corroborates the pathophysiological process of DM1 [17,20]. From a metabolic point of view, it can contribute to the development of insulin resistance, obesity, and atherosclerotic vascular lesions in T1DM patients, showing a negative correlation with the level of high-density lipoprotein cholesterol [17,21,22].

The potential role of TNF-α in initiating the apoptotic process and its cytotoxic response in β cells was already reported. Still, it is also essential to know its role during the chronic evolution of the disease [18].

Diabetes and diabetes-related complications in COVID-19 patients are primarily due to the acute illness caused during the SARS-CoV-2 infection followed by the release of glucocorticoids, catecholamines, and pro-inflammatory cytokines, which were shown to drive hyperglycemia positively [23].

Cytokines were studied in many pathologies, and their understanding was a therapeutic focus [24,25]. Understanding how cytokines are expressed in DM1 patients will help us to unravel this inflammatory network that affects this pathology. There are few conflicting data on the role of IL-10 and TNF-α in the pathogenesis and progression of T1DM. For these reasons, we aimed to investigate the gene expression of IL-10 and TNF-α in TDM1.

## 2. Materials and Methods

### 2.1. Research Design

It was a cross-section observational study in which patients with T1DM of both sexes were consecutively selected in the Endocrine Outpatient or the Emergency Service of the Hospital das Clínicas da Faculdade de Medicina de Marilia Sao Paulo, Brazil, from February 2018 to August 2019.

The protocol of this study was established based on the guidelines presented by the International Society for Pediatric and Adolescent Diabetes (ISPAD) [26].

The minimum sample size was estimated at 73 sample elements, considering a large effect size (0.40), a type I error margin (α) of 5%, a study power of 80%, and four (4) groups of comparison using a one-way ANOVA. Sample size calculation was performed using the G*Power software, version 3.1.9.2 (Franz Faul, Universität Kiel, Germany). The final sample consisted of 107 sample elements.

### 2.2. Ethics

The Marilia School approved this study by Medicine Research Ethics Committee, and an informed consent form was obtained from all participants (Prot. Number 2.444.417, approval date: 6 December 2017).

### 2.3. Participants and Eligibility

All the included patients were T1DM according to the definition of ADA [27]. None of them used glucocorticoids, other immunosuppressive drugs, or illicit drugs. Patients with other active autoimmune and/or non-autoimmune inflammatory diseases and a current or past history of malign neoplasia were also excluded. The control group consisted of healthy individuals with normal fasting plasma, defined as fasting plasma glucose < 100 mg/dL and HbA1c < 5.7%, matched for age, gender, and body mass index (BMI). They were in good general health condition and were not taking any medication.

### 2.4. Groups

The reference criteria for defining patients with diabetic ketoacidosis were those proposed by the International Society for Pediatric and Adolescent Diabetes (29) during the study period. To form comparison groups, group 1 (diabetic ketoacidosis) was included to distinguish patients who entered the study during an event of diabetic ketoacidosis. Groups 2 and 3 were patients who did not experience ketoacidosis in the past 12 months but differed in their glycemic control state, as measured by their HbA1c levels. Group 2 included patients with inadequate glycemic control, with HbA1c values > 8%, while group 3 included patients with better glycemic control, with HbA1c values < 8%. It is important to note that all patients who were hospitalized with ketoacidosis during the study period had a diagnosis of TDM1, and no patients were excluded.

The participating individuals were divided into four groups:

1. Group 1: patients with T1DM during an episode of ketoacidosis, admitted to the emergency service up to 72 h after the event;
2. Group 2: T1DM patients with HbA1c ≥ 8%;
3. Group 3: T1DM patients with HbA1c < 8%;
4. Group 4: a control group with healthy participants (without TDM1).

### 2.5. Measurements

Patients and controls were submitted to clinical and biochemical evaluations. Bodyweight and height were measured with empty bladder while they were wearing light clothing and without shoes. BMI was evaluated by dividing the weight (kg) by the height in centimeters squared.

Information regarding the age of diagnosis and time since diagnosis in months was collected for patients in groups 1, 2, and 3. Age of diagnosis refers to the age at which the patient was diagnosed with TDM1. Time since diagnosis indicates the duration of time elapsed since the patient’s diagnosis of TDM1, considering the period during which the data was collected.

Blood samples were investigated for biochemical parameters, and fasting blood was taken for immediate and future analyses in the local laboratory, where they were stored at −20 °C.

Plasma glucose was measured by the in vitro test for the quantitative determination of glucose in serum, using Roche/Hitachi Cobas c. Systems, Reference enzyme method with hexokinase, Roche Diagnostics, USA, with a considerable range of normality ≤ 99 mg/dL.

HbA1c was measured by the method of high-performance liquid chromatography (HPLC), using only freshly collected capillary blood, whole venous blood with lithium heparin, K2, or K3 EDTA, with the value used in the HbA1c test to diagnose diabetes, with a threshold ≥ 6.5%. C-peptide was performed by electrochemiluminescence assay (electrochemiluminescence immunoassay or “ECLIA”) with a lower detection limit: 0.003 nmol/L (0.010 ng/mL), Roche Diagnostics, USA Microalbuminuria was performed using the Immunoturbidimetric Assay method, in a 12 h urinary sample with a measurement range 3.0–200 mg/L (0.05–3.10 µmol/L or 0.3–20.0 mg/dL), Roche Diagnostics brand the USA.

### 2.6. RNA Extraction

Samples of venous blood were collected (4 mL) from all individuals and transferred to tubes containing K3EDTA (Greiner Bio One Vacuette^®^ GmbH, Kirchdorf an der Krems, Austria) as an anticoagulant. QIAamp^®^ RNA Blood Mini Kit–cat. n. 52304 (QIAGEN, Hilden, Germany) was used for total RNA extraction of peripheral blood, according to instructions from the manufacturer. RNA samples were quantified using NanoDrop ND-2000 Spectrophotometer (Thermo Scientific, Waltham, MA, USA) for subsequent storage at −20 °C up to the moment of its use.

Gene expression was measured by RT-qPCR according to Livak et al. [28] on the Applied Biosystems 7500 Fast Real-Time PCR system (Applied Biosystems™, Waltham, MA, USA), using TaqMan Gene Expression Assays (FAM™ dye-labeled MGB probes), for targets genes IL-10 (assay id: Hs00240518_m1), TNF-α (assay id: Hs00240982_m1), and endogenous control B2M (assay id: Hs99999907_m1), and ADH (assay id: Hs03929097_g1).

The amplification curve of each group was drawn, and the cycle threshold (CT) values were obtained for all genes and set threshold values uniformly for all assays. All reactions were performed in duplicate and replicated with a standard deviation (SD) higher than 0.5 for the CT value that was repeated or excluded from the analysis. Relative quantification (RQ) was analyzed using the comparative CT method (2−ΔΔCT). All CT values were obtained by 7500 software 2.0, and these data were exported to Excel software (Microsoft, Redmond, WA, USA) according to the study of Livak et al. [28].

### 2.7. Diabetes Definitions Criteria

Diabetes was defined based on plasma glucose criteria according to the current clinical practice recommendations of the American Diabetes Association (ADA) [27]. T1DM was designated when these criteria were associated with typical manifestations of the disease, such as sudden onset, classic symptoms such as polyuria, polydipsia, and polyphagia, previous or current episode of ketoacidosis, or autoimmunity confirmed by a positive antibody involved in the pathogenesis of T1DM, such as anti-glutamic acid decarboxylase (GAD65) antibody, anti-zinc transporter 8 (ZnT8A), anti-islet antibody, and anti-insulin antibody in an insulin-dependent patient [4].

Diabetic ketoacidosis (DKA) was defined by the presence of hyperglycemia (plasma glucose > 200 mg/dL), venous pH < 7.3 or serum bicarbonate < 15 mmol/L, and by the presence of ketonemia (serum ß-hydroxybutyrate ≥ 3 mmol/L) and/or ketonuria [29].

Poor metabolic control was defined by an average HbA1c above 8% (obtained by three determinations in the last 12 months) [30].

### 2.8. Statistical Analysis

Qualitative variables were described by the distribution of absolute (N) and relative (%) frequency. The association between qualitative variables was performed using the chi-square test. Quantitative variables were described by mean and standard deviation (SD). The normality distribution was verified by the Komolgorov–Smirnov test with Lilliefors correction. To compare two independent groups, the Student t-test or the Mann–Whitney nonparametric test was performed. For comparison of means among independent groups, a one-way ANOVA or Kruskal–Wallis nonparametric test was used, followed by the post hoc test with Holm–Sidak correction when necessary. Pearson or Spearman correlation analysis was used to analyze the relationships between quantitative variables. The significance level adopted was 5% (*p* ≤ 0.05), CI95%, and data were analyzed in the SPSS software (24.0 version, IBM, Armonk, NY, USA).

## 3. Results

The main characteristics of TDM1 and controls are shown in Table 1. Seventy-seven T1DM patients and 30 healthy controls enrolled in this study; 52.3% were female, and ages ranged from 1 to 21 years. There were no significant differences in terms of age, sex, z-height, and z-BMI among the groups. As expected, the fasting plasma glucose (FPG) and HbA1c levels were significantly higher, and C-peptide levels were significantly lower among the patients compared to control participants.

Table 2 shows the parameters among the three TDM1 patients and control groups. Group 2 and 3 patients showed a significantly higher mean age (*p* = 0.01) and diagnose time (*p* = 0.005), as well as FPG (*p* < 0.001) and C-peptide (*p* < 0.001) levels, but lower basal and mean HbA1c (*p* < 0.001) compared to those of group 1. Significant differences were observed between groups for the expression of IL-10 RQ (*p* < 0.001), with significantly higher values in group 1 compared to the other groups 2, 3, and controls. In contrast, TNF-α RQ gene expression was significantly higher only in group 3 compared to controls. No apparent difference was found in sex, z-height, z-BMI, and diagnostic age among the groups.

**Figure 1:**
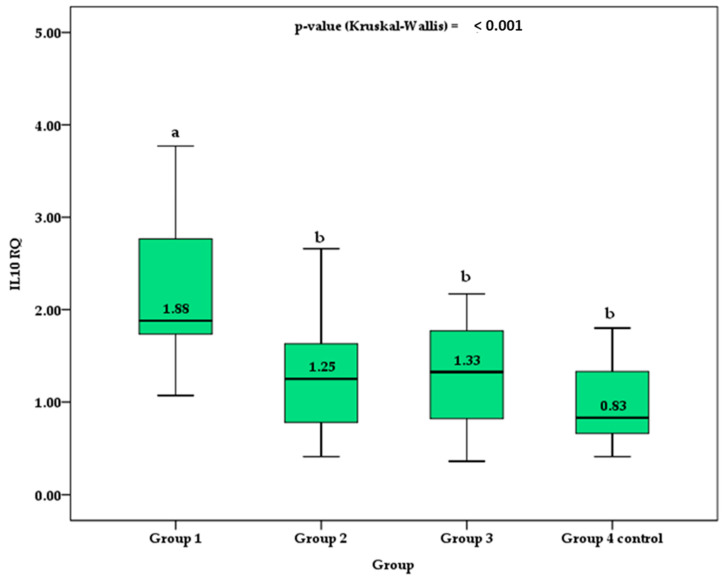
Comparison of the median and interquartile range (box) of IL-10 RQ between groups. Different superscript letters indicate a significant difference between the means by the post hoc Holm–Sidak test for *p*-value < 0.050. Different superscript letters indicate a significant difference between the means by the post hoc Holm–Sidak test for *p*-value < 0.050.

In Figure 1, the box plot depicts the median IL-10 RQ values between groups. Group 1, which represents patients diagnosed with TDM1 who were admitted to the emergency room with an episode of ketoacidosis, had median values higher than those of group 2, group 3, and control group 4. Although control group 4 presented lower IL-10 RQ values than groups 2 and 3, no significant difference was verified by the post hoc Holm–Sidak test.

**Figure 2:**
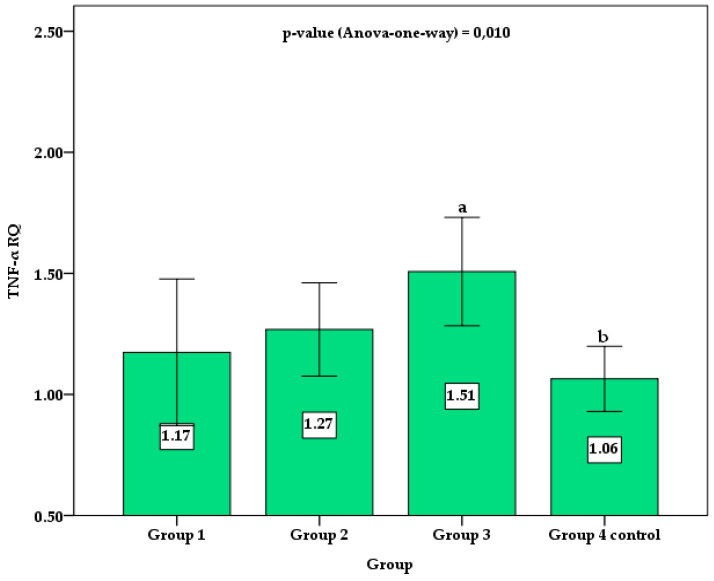
Comparison of the mean and 95% confidence interval (error bar) of TNF RQ between groups. Different superscript letters indicate a significant difference between the means by the post hoc Holm–Sidak test for *p*-value < 0.050.

Figure 2 shows the column chart with the mean and 95% confidence interval (95%CI) for the TNF RQ values between groups. Control group 4 showed statistically lower values than group 3. Although group 3 showed mean values of TNF RQ higher than groups 1 and group 2, no statistically significant difference was verified by the post hoc Holm–Sidak test. No significant difference was observed between control group 4 and groups 1 and 2 of patients with TDM1.

We performed correlation analyses to investigate potential relationships among the studied interleukin’s relative gene expression and the studied quantitative variables. The relative gene expression value of IL-10 (*p* = 0.001) and TNF-α (*p* = 0.015) was significantly higher among participants with T1DM than controls. There was a positive correlation between IL-10 RQ and HbA1c (*p* < 0.001), a negative correlation between IL-10 and the age of subjects with diabetes (*p* = 0.029), and time of diagnosis of the disease (*p* = 0.036). TNF RQ positively correlated with age in all groups (*p* = 0.001). Although a significant correlation was observed between IL-10 RQ and age, time and diagnosis, HbA1c and glycemia, as well as between TNF RQ and age, the correlation coefficient values suggested a moderate to a low degree of relationship (Table 3).

## 4. Discussion

The development of DM1 requires a series of physiological changes, ranging from genetic predisposition and imbalance of effector and regulatory T cells with loss of central and/or peripheral tolerance to synergistic or antagonistic interaction between cytokines, generating an inflammatory process that culminates in the destruction of the β cell and onset of diabetes [20,31].

In recent years, the role of immune checkpoints in the treatment of cancer was increasingly recognized, but unfortunately, little attention was paid to the significant role they played both in the development of secondary diabetes with immune checkpoint inhibitors and the treatment of T1D, such as cytotoxic T-lymphocyte antigen 4(CTLA-4), programmed cell death protein-1(PD-1), lymphocyte activation gene-3(LAG3), programmed death ligand-1(PD-L1), and T-cell immunoglobulin mucin protein-3(TIM-3). Immune checkpoint inhibitors related to diabetes, similar to T1D, are induced by severe endocrine toxicity with immune checkpoint inhibitors. Numerous treatment measures showed excellent efficacy for T1D by regulating diverse immune checkpoint molecules, including co-inhibitory and co-stimulatory molecules. Thus, targeting immune checkpoint molecules may exhibit potential for T1D treatment and improve clinical outcomes [32].

Our results showed that patients with diabetes presented higher IL-10 and TNF-α gene expression compared to those without diabetes, suggesting an increased inflammatory activity. As HbA1c levels decrease, there is a change in a cytokinetic profile with a decrease in the expression of IL-10, showing a compensatory cytokine response of defense [33]. Therefore, the cytokine pattern found in this study would be a response to the glycemic damage, varying according to the metabolic state and consequent cell aggression [34,35]. On the other hand, before the increase in IL-10, suppression of TNF-α could occur [11].

Thus, the increase in IL-10 expression in more severe cases of diabetes could be a reaction to the metabolic stress resulting from ketoacidosis or hyperglycemia [36,37]. The modulation of IL-10 could be a target of intervention once there is still no cure for T1DM.

Our results also showed that IL-10 expression decreases over the time of T1DM diagnosis, and there is an increase in the levels of TNF-α, changing the cytokine profile of cytokines from anti- to pro-inflammatory.

Although the role of cytokines is unclear and complex, they were associated with the pathogenesis of diabetes due to a complex multicellular interaction that was observed between β cells and immune cells. In DM1, increased and progressive lymphocytic infiltration in the pancreatic islets was observed by immune system cells, mainly represented by macrophages, CD4+, and CD8+T cells resulting in inflammatory infiltration and depletion of insulin production and the death of β-cells [18,38,39,40].

Diabetes patients evolve heterogeneously, some more aggressive and others milder. One of the factors that may be related is the degree of this cellular destruction, and the residual insulin production, measured through the C-peptide, can contribute to better glycemic control [41].

Some studies showed that despite IL-10 presenting impressive immunosuppressive actions, its role in modulating the disease progression is controversial [12]. Lu et al. [40] and Robert et al. [42] proposed that in animal models, the intervention targets that stimulate IL-10 improved the function of β-cells and inhibited the progression of insulitis [40,42,43]. Some animal models suggested that intervention targets that stimulate IL-10 improve β-cell function and inhibit the progression of insulitis [44]. In humans, Rapoport et al. and Kleffel et al. [45,46] showed that IL-10 plays a protective role once it is associated with disease attenuation. The effects of IL-10 in T1DM may be explained by the suppression of IL-2 and INF-γ and an increase in the Treg stimulation. Moreover, IL-10 can be linked to a tolerant state of immature dendritic cells and regulatory IL-10-releasing B cells in T1DM subjects [42,46].

On the other hand, IL-10 can exhibit a pathogenic action at the beginning of the disease, and its local release can accelerate the onset and increase the prevalence of diabetes [47,48,49,50]. These complex and dual actions show both immunosuppressive and stimulatory effects of IL-10. Indeed, type 1 interferon signals, which trigger T1DM in susceptible subjects, can inhibit IL-10 signalization via the JAK/STAT pathways in effector T cells and Treg cells, modifying or modulating the behavior of these cells in T1DM. TNF-α, together with the augmented expression of IL-1*β* and interferon-gamma (IFN-*γ)*, acts in synergy during the inflammation of *β*-cells, resulting in the activation of nuclear factor-*κ*B that can be activated by several stimuli such as TNF-α, IL-1, advanced glycation end products, and Toll-like receptors [51].

With the progression of the disease, there may be an adaptation to this inflammatory process, and the decrease in IL-10 would allow the increase in the levels of TNF-α, which is an essential factor in maintaining and expanding inflammation in chronic diseases [36]. In turn, this cytokine affects T cells due to the increase in the proliferation and production of IFN-γ. The combination of these two cytokines in the initial stages of the disease leads to the death of β cells through IL-1 regulation, nitric oxide production, and caspase activation. During apoptosis, cells release a plethora of intracellular components and expose membrane components. Consequently, a removal process of necrotic and apoptotic body cells by phagocytic cells would begin, with leukocyte recruitment and TNF-α as one of its agents. Considering the repair stage by activated macrophages, there is the release of substances that promotes tissue repair, such as IL-10 and TGF-β [52,53,54].

An in vitro study showed that targeted overexpression of TNF-α in pancreatic cells of transgenic mice augmented the progression of diabetes [55]. Another investigation performed in adoptive transfer models suggested that TNF-α plays a crucial role in Th1 and Th2 cells during diabetes induction [56]. The anti-TNF administration to the newly onset T1DM mice model restored homeostasis in the glycemia and self-tolerance, preventing the disease progression [57,58]. On the other hand, the treatment with anti-TNF-α at four weeks of age or later accelerated the progression of the disease [59]. Still, the systemic administration of this cytokine protected against T1DM [60]. These studies showed that TNF-α may work as inhibiting or inducing inflammatory pathways. The direction of the answer is probably related to some factors that may include the stage of the disease and genetic susceptibility [20,61].

These findings could explain why, in moments of increased oxidative stress (such as ketoacidosis), there would be an increase in TNF-α and IL-10 levels. Moreover, IL-10 decreases in patients who were stable for a long time due to reduced oxidative stress and the inflammatory process in patients with stable glycemic control. For these reasons, a new possibility of a therapeutic approach for patients with diabetes could be based on immunogenic therapy, as other studies already suggested [61,62].

Since IL-10 is a potent anti-inflammatory cytokine and inflammatory aTNF-α, the chronicity of the disease may be linked to worsening the inflammatory profile in these patients. With the use of cytokines in several therapeutic areas of science, it is necessarily better to understand the gene expression of cytokines in this pathology. This study starts with two cytokines, one anti- and one pro-inflammatory.

Patients with DM 1, although they are subclassified as DM1A and DM1B, clinically evolve differently from the point of view of both acute complications, such as ketoacidosis, as well as chronic micro and macrovascular complications. This difference may be related to genetic predisposition, beta cell insulin reserve, and individual inflammatory activity [63].

Another fact that draws attention is the stages of DM1 (stage 1: multiple islet autoantibodies, normal blood glucose, presymptomatic; stage 2: multiple islet autoantibodies, abnormal glucose tolerance, usually pre-symptomatic; stage 3: blood glucose above ADA diagnostic threshold; stage 4: established T1D). The progressive acceleration of cell destruction leads to phase change and there is an individual variation in the time it takes to pass from one phase to another, suggesting an immunological process specific to each individual. More than 70 genetic variants were associated with DM1 through genomic studies, the Loci HLA DR and HLADQ associate half the risk, but an individual predisposition leads to disease progression, time of presentation, and evolution with complications. Among the factors that may contribute to the different presentations, there was speculation about numerous motivations such as microbiota, vitamin D, and viral infections, highlighting the effects that environmental exposure plays in the pathogenesis of DM1, with a possible opening up in the individual inflammatory expression [26,64,65].

## 5. Conclusions

The role of cytokines and their gene expression in DM1 is still obscure and complex without complete clarity on the unique role of inflammatory patterns, many having a double effect on the development of the disease. There is a way to go and that knowledge in this area will help us understand the pathophysiology and evolution of the disease. However, we need to evaluate the expression of other cytokines to understand the cytokine networks involved in the pathogenesis of DM1 and their interrelation with factors such as intestinal microbiota, endocrine disruptors, and plasticity.

Current T1DM treatment is based mainly on exogenous insulin administration, showing the need for other therapies that could improve clinical outcomes and the patient’s quality of life. To the best of our knowledge, we showed, for the first time, the correlation between T1DM and gene expression of IL-10 and TNF-α.

## Figures and Tables

**Table 1:** Demographic and laboratory parameters for T1DM patients and controls.

Parameters	T1DM *n* = 77	Controls *n* = 30	*p*-Value
Age (years)	13.0 (3.8)	12.6 (3.8)	0.600
Female, *n* (%)	33 (42.9)	17 (56.7)	0.200
z-Height	0.15 (1.0)	0.57 (1.1)	0.060
z-BMI (kg/m^2^)	0.49 (0.9)	0.85 (1.1)	0.900
FPG (ng/dL)	210 (134)	83.9 (5.7)	<0.001
HbA1c (%)	9.0 (2.3)	4.9 (0.3)	<0.001
C-peptide (nmol/L)	0.32 (0.5)	2.34 (0.8)	<0.001

BMI, body mass index; FPG, fasting plasma glucose; HbA1c, glycated hemoglobin. Values were expressed as mean (SD), unless noted otherwise.

**Table 2:** Comparison of mean and standard deviation (SD) of quantitative variables between study groups.

	Group 1 (TDM1) *n* = 15	Group 2 *n* = 32	Group 3 *n* = 30	Controls *n* = 30	*p*-Value
Age (years)	10.1 (4.0) ^a^	13.5 (2.9) ^b^	13.8 (4.0) ^b^	12.6 (3.8)	0.0100 *
Female, *n* (%)	7 (46.7)	17 (53.1)	9 (30.0)	17 (56.7)	0.850
z-Height	0.13 (1.3)	0.09 (1.0)	0.22 (0.9)	0.58 (1.1)	0.290
z-BMI (Kg/m^2^)	0.11 (1.29)	0.53 (0.9)	0.59 (0.94)	0.85 (1.14)	0.160
Age of diagnosis of TDM1 (months)	94.6 (41.10)	98.7 (38)	97.3 (41.9)		0.940
TDM1 diagnostic time (months)	27.0 (39.5) ^a^	65.9 (38.1) ^b^	67.0 (61.9) ^b^		0.005 †
FPG (ng/dL)	389.2 (127.5) ^a^	190.8 (111.5) ^b^	141.4 (65.5) ^b^	83.9 (5.7) ^c^	<0.001 †
Basal HbA1c (%)	11.8 (2.1) ^a^	9.7 (1.5) ^b^	6.8 (0.8) ^c^	4.9 (0.4) ^d^	<0.001 †
Mean HbA1c (%)	10.3 (2.3) ^a^	9.3 (1.3) ^a^	7.1 (1.2) ^b^		<0.001 †
C-Peptide (nmol/L)	0.31 (0.2) ^a^	0.15 (0.2) ^b^	0.45 (0.7) ^b^	2.23 (0.8) ^c^	< 0.001 †
IL10 RQ	2.63 (1.62) ^a^	1.3 (0.62) ^b^	1.30 (0.53) ^b^	1.05 (0.67) ^b^	<0.001 †
TNF-α RQ	1.17 (0.55)	1.27 (0.53)	1.50 (0.53) ^a^	1.06 (0.36) ^b^	0.010 *

BMI, body mass index; FPG, fasting plasma glucose; HbA1c, glycated hemoglobin; IL-10 RQ, onterleukin-10 gene expression relative quantification; TNF-α RQ, tumor necrosis factor-alpha gene expression quantification. Group 1 TDM1 with ketoacidosis, Group 2 T1DM patients with HbA1c ≥ 8%, Group 3: patients with T1DM with HbA1c < 8%, and Group 4: control group. * Significant difference between the ANOVA test groups; † significant difference between the Kruskal–Wallis nonparametric test groups; different superscript letters indicate a significant difference between the means by the post hoc Holm–Sidak test for *p*-value < 0.050.Values expressed as mean (SD) unless noted otherwise.

**Table 3:** Gene expression of IL-10 and TNF-α according to some parameters of the patients included in the study.

	IL10 RQ		TNF RQ	
	r	*p*-Value	r	*p*-Value
Age (years)	−0.248	0.029 *	0.29	0.010 *
Diagnostic time (months)	−0.238	0.036 *	0.158	0.170
z-BMI kg/m^2^	−0.094	0.421	−0.002	0.986
Fasting blood glucose (mg/dL)	0.272	0.016 *	−0.152	0.186
% HbA1c	0.226	0.048 *	−0.174	0.129
C-Peptide	0.247	0.069	0.063	0.648
Gender (1 = male; 2 = female)	−0.089	0.441	−0.096	0.405

* Significant correlation by Spearman’s nonparametric test for *p*-value ≤ 0.05. r = correlation coefficient.

## Data Availability

Not applicable.
